# Supplementary material for: Identification of Chromosomal Regions and Candidate Genes for Round leaf Locus in Cucumis melo L
Source: Plants (Basel). 2024 Apr 18;13(8):1134. doi: 10.3390/plants13081134 (PMC11054961; doi:10.3390/plants13081134)
Supplement: Supplementary file 1 [file plants-13-01134-s001.zip › Supplementary Figures.pdf]

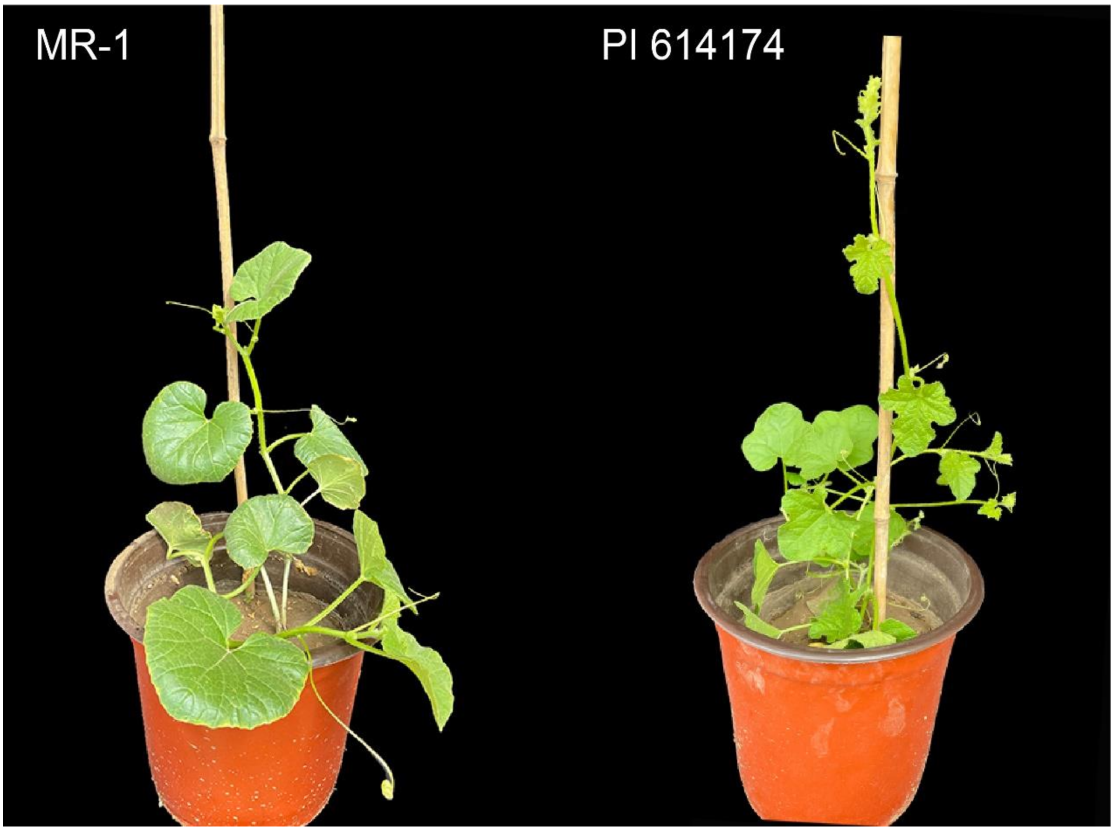

Figure S1. Whole-plant diagrams of the development of MR-1 and PI 614174.

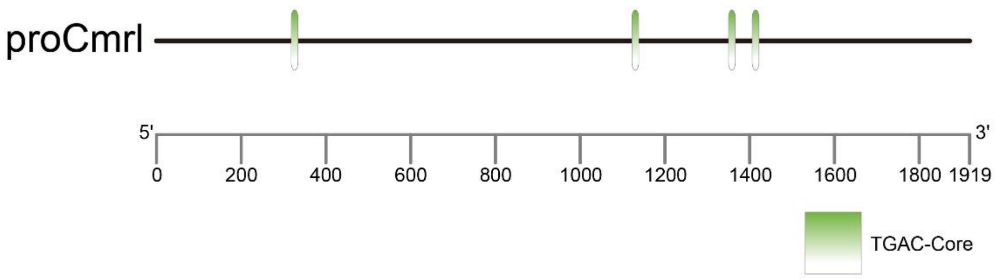

Figure S2. Distribution of TGAC core *cis*-acting elements on the *MELO3C019152.2* promoter.
